# Necroptosis as a potential therapeutic target in multiple organ dysfunction syndrome

**DOI:** 10.18632/oncotarget.18252

**Published:** 2017-05-29

**Authors:** Yao-Li Cui, Li-Hua Qiu, Shi-Yong Zhou, Lan-Fang Li, Zheng-Zi Qian, Xian-Ming Liu, Hui-Lai Zhang, Xiu-Bao Ren, Yong-Qiang Wang

**Affiliations:** ^1^ Department of Lymphoma, Tianjin's Clinical Research Center for Cancer and Key Laboratory of Cancer Prevention and Therapy, Tianjin Medical University Cancer Institute & Hospital, National Clinical Research Center for Cancer, Tianjin 300060, China; ^2^ Department of Intensive Care Unit and Key Laboratory for Critical Care Medicine of The Ministry of Health, Emergency Medicine Research Institute, Tianjin First Center Hospital, Tianjin 300192, China; ^3^ Department of Biotherapy, Key Laboratory of Cancer Immunology and Biotherapy and Key Laboratory of Cancer Prevention and Therapy, Tianjin's Clinical Research Center for Cancer, Tianjin Medical University Cancer Institute & Hospital, National Clinical Research Center for Cancer, Tianjin 300060, China

**Keywords:** multiple organ dysfunction syndrome, necroptosis, necrosome, high-mobility group box 1, mixed-lineage kinase domain-like

## Abstract

**Purpose:**

To investigate how necroptosisis, i.e. programmed necrosis, is involved in MODS, and to examine whether Nec-1, a specific necroptosis inhibitor, ameliorates multiorgan injury in MODS.

**Experimental Design:**

A model of MODS was established in six-week old SD rats using fracture trauma followed by hemorrhage. Control animals received sham surgery. Cell death form and necrosome formation were measured by fluorescence-activated cell sorting and western blotting. MODS rats were randomly assigned to receive Nec-1 or saline with pretreatment and once daily. The first end-point was 72 hours survival. Organ injury and dysfunction, inflammatory cytokine levels, and necroptotic execution protein expression were also recorded.

**Results:**

Organ injury and dysfunction were significantly more severe in the MODS group than the sham group (all *p*<0.01). Furthermore, MODS-induced liver, lung and kidney tissue injury was characterized by necroptosis rather than apoptosis, and accompanied by necrosome formation. Compared to MODS group, Nec-1 administration significantly improved 72 hours survival (*p*<0.01). Nec-1 administration significantly reduced necroptosis-induced liver, lung and kidney injury and dysfunction, inhibited inflammatory cytokines production, inhibited release of necroptotic execution proteins such as high-mobility group box 1 and mixed-lineage kinase domain-like protein pseudokinase in MODS rats (all *p*<0.01).

**Conclusions:**

These results suggest that necroptosis is involved the pathology of MODS. Further, a necroptotic inhibitor Nec-1 may be considered as an adjunct treatment for MODS.

## INTRODUCTION

MODS describes the progressive dysfunction of two or more organ systems following an acute threat to systemic homeostasis. MODS is the leading cause of mortality in critically ill patients [1], and in most cases occurs secondary to severe sepsis or septic shock, trauma, neoplastic diseases, or SIRS [2]. To improve the prognosis of this disease, the pathological and physiological processes comprising MODS have been investigated, however, its complex multifactorial pathology remains poorly understood [3–5].

In 1991, a joint conference committee of the American College of Chest Physicians and the Society of Critical Care Medicine described SIRS and the corresponding compensatory anti-inflammatory responses [6] in which a cascade of effects arise from an imbalance between pro- and anti-inflammatory mediators, leading to the development of MODS [7–9]. However, no effective treatments or preventive measures have been found to successfully target inflammation in MODS. Therefore, understanding the molecular mechanisms underpinning this syndrome may inform potential clinical treatment strategies.

Necrosis has been considered a non-programmed form of cellular injury and premature death; however, it was recently recognized that one form of cell death morphologically classified as necrosis could also be regulated in a programmed manner via defined signal transduction pathways. This process was termed necroptosis, which is dependent RIP1, RIP3, and a MLKL form a multiprotein complex called a necrosome [10]. Accumulating evidence indicates that necroptosis is involved in the regulation of lethal SIRS [11–14], ischemia-reperfusion injury [15], and traumatic injury [16]. Therefore, elucidating the molecular mechanisms underpinning necroptosis may inform development of novel treatment strategies for MODS.

In recent years small molecule necroptosis inhibitors have proved to be both effective tools with which to study cell death, and to have the potential to serve as therapeutic agents for necroptotic diseases. For example, Nec-1 was reported as the first inhibitor of necroptosis, and exhibited therapeutic potential [17–18]. In this study, we found that necroptosis is involved the pathologic process of MODS, and further that Nec-1 may represent a clinically efficacious adjunct treatment in MODS.

## RESULTS

### Successful establishment of MODS rat model

**Figure 1:**
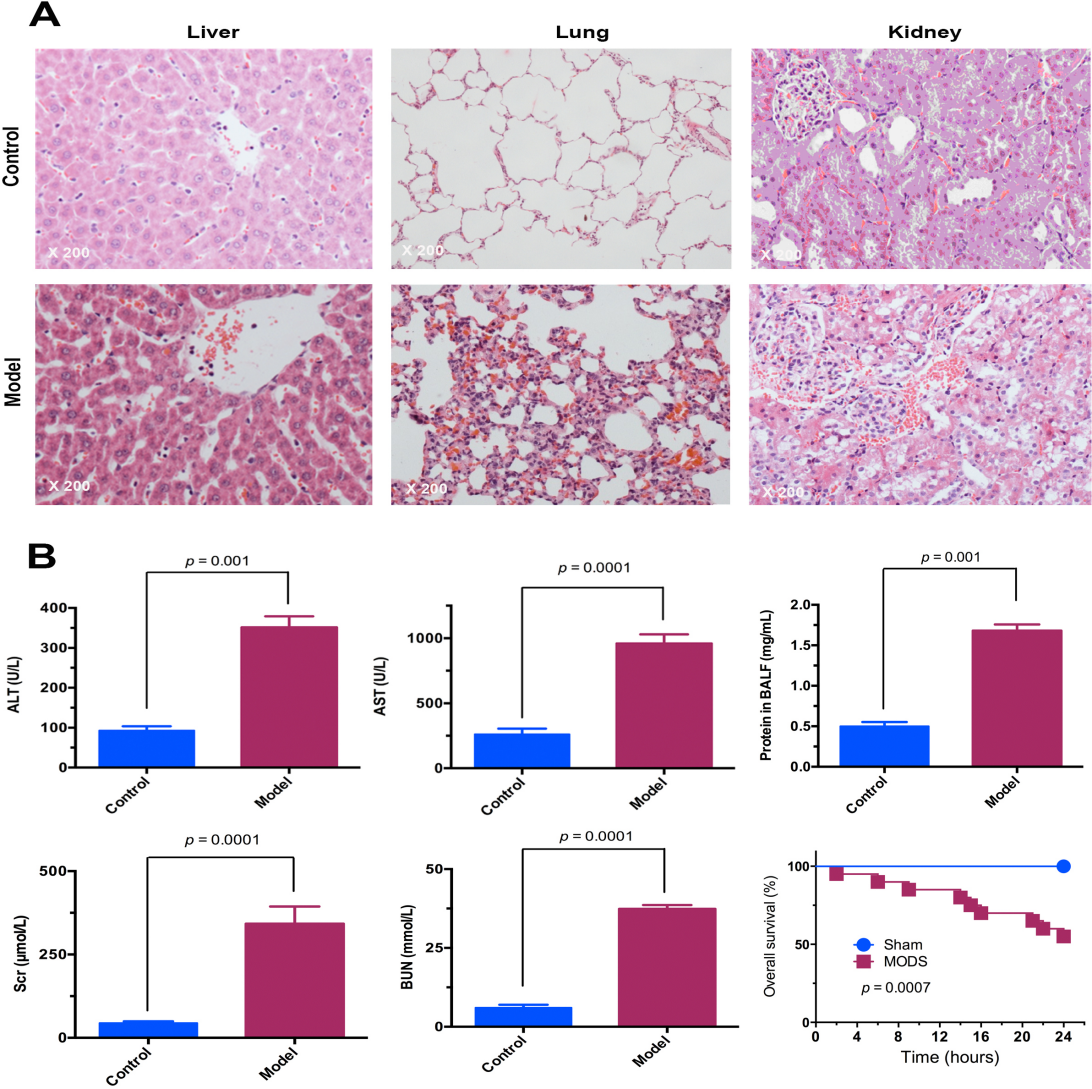
The MODS rat model was successfully established **(A)** Pathological changes in the liver, lung, and kidney of MODS and sham group rats. Tissues were stained with HE and observed under light microscope (magnification, ×200). **(B)** Multiorgan function marker levels in MODS and sham groups: liver function markers; lung function marker; kidney function markers, and survival of MODS and sham group rats 24 hours.

A SD rat model of MODS was successfully established using fracture trauma followed by hemorrhage. MODS was manifested in significantly more severe multiorgan injury than sham surgery at 12 hours (Figure 1A and 1B). In the MODS group, levels of multiorgan dysfunction markers were significantly higher than in the sham group at 12 hours (all *p*<0.01). Furthermore, the 24 hours survival rate in the MODS group was 60%, while no animals in the sham group died (*p*<0.01).

### MODS-induced multiorgan necroptosis dominates over apoptosis

**Figure 2:**
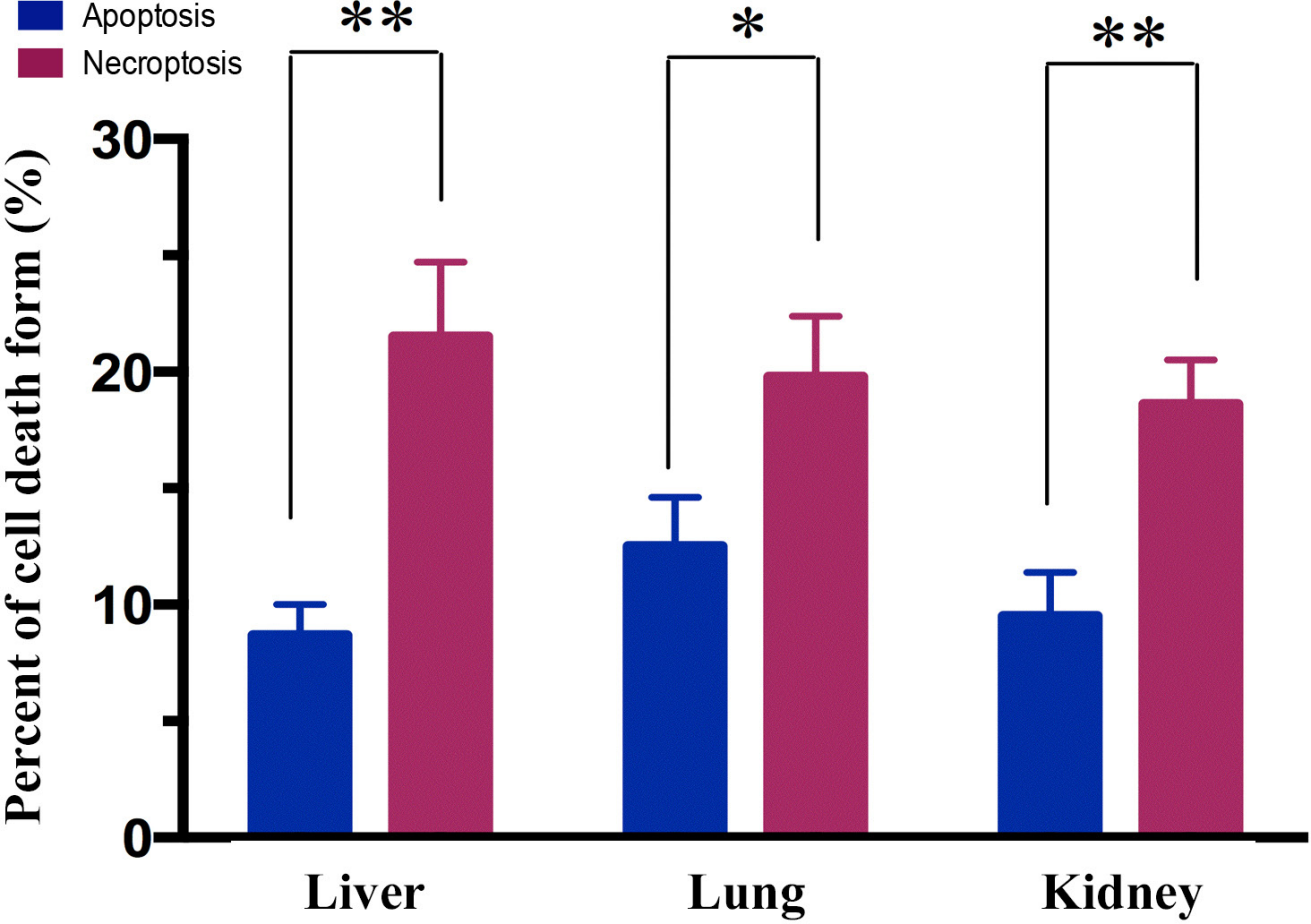
Necroptosis dominates over apoptosis in MODS rats Rats from MODS group was sacrificed after inoculation; their tissues were obtained. Annexin V/PI staining was assessed by flow cytometry to assess the form of cell death in multiorgan. Apoptosis: Annexin-V positive cells; necrosis: PI positive and Annexin-V negative cells (mean ± standard deviation). * *p*<0.05, ** *p*<0.01.

In order to determine which form of cell death was involved in MODS, we measured the percentage of apoptotic and necroptotic (necrotic) cells at 12 hours after MODS using flow cytometry. We found that in MODS necroptosis dominates over apoptosis in liver, lung, and kidney at 12 hours (Figure 2, all *p*<0.05).

### Necrosome formation involved in MODS-induced necroptosis

**Figure 3:**
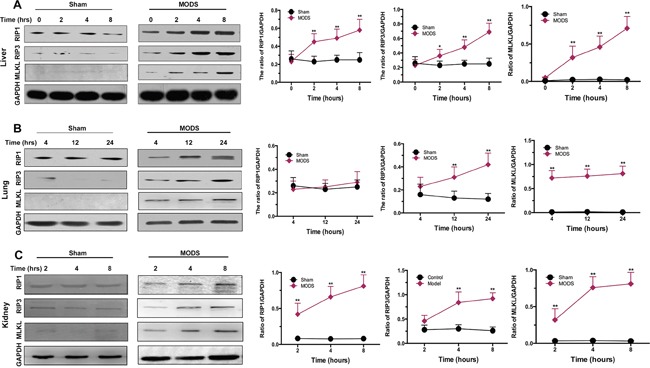
Necrosome formation in MODS-induced necroptosis Rats from the sham and MODS groups were sacrificed; organ tissues were obtained. **(A-C)** Expression of RIP1, RIP3, and MLKL protein was evaluated by western blotting in liver, lung, and kidney. Relative protein expression levels were calculated relative to GAPDH. Data are expressed as mean ± standard deviation. * *p*<0.05 vs. the sham group, ** *p*<0.01 vs. the sham group.

To investigate the expression of components of the necrosome in MODS, we measured RIP1, RIP3, and MLKL expression in the liver, lung, and kidney of MODS model and sham rats. We found that RIP1, RIP3 and MLKL were expressed significantly more highly in the liver, lung and kidney of MODS animals than sham-group animals in a time-dependent, while RIP1 expression did not difference significantly between two groups in the lung (all *p*<0.05, Figure 3A-3C).

### Nec-1 administration improves the prognosis of rats with MODS

**Figure 4:**
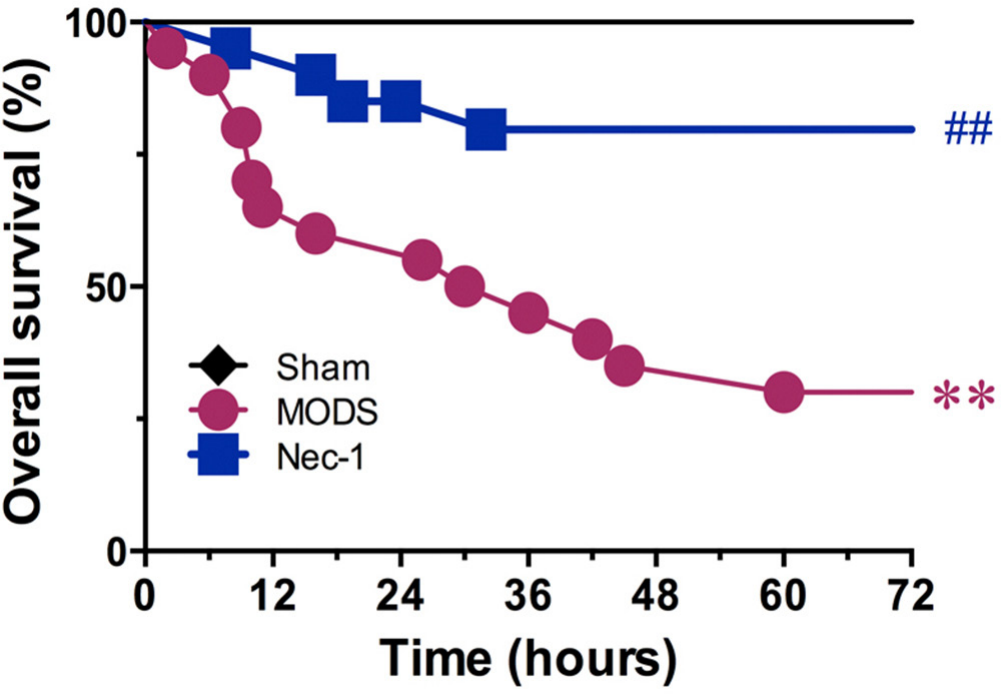
Effect of Nec-1 on MODS survival (25 animals per group) ** *p*<0.01 vs. the sham group, ^#^ *p*<0.05 vs. the MODS group, and ^##^*p*<0.01 vs. the MODS group.

Seventy-two hours after surgery, all sham group animals survived, while the survival rate in the MODS group was only 65% at 24 hours and 35% at 48 hours after surgery. Log-rank analysis of the 72 hours survival curves demonstrated that administration of Nec-1 improved the survival rate in the MODS group to 85% at 24 hours and 80% at 72 hours after surgery (all *p*<0.01, Figure 4).

### Nec-1 administration reduces necroptosis-induced liver injury

**Figure 5:**
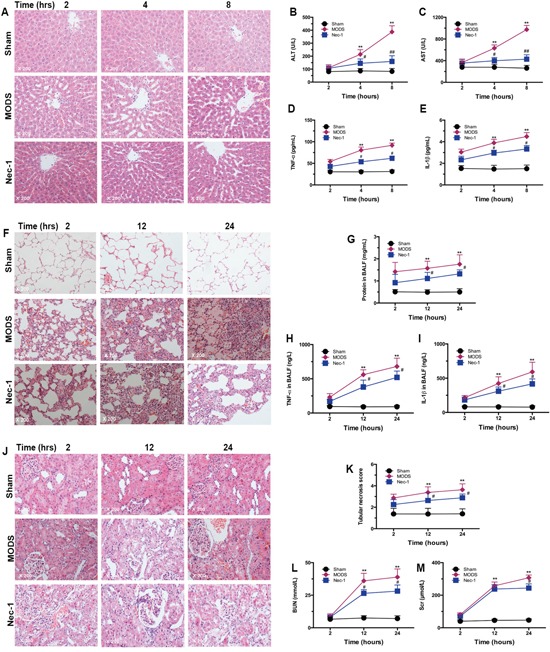
Nec-1 treatment significantly reduced necroptosis-induced multiorgan injury and dysfunction Histological examination, biochemical analysis, and markers of systemic inflammation were assessed. Rats were sacrificed after inoculation, and organ tissues, serum, and BALF were obtained (**A, F**, and **J**). Liver, lung, and kidney tissues were dissected and fixed in 4% paraformaldehyde. Paraffin-embedded tissue sections were stained with HE and examined by light microscopy. Images are representative of each group in three separate experiments (original magnification ×200). **(A)** Liver; **(F)** lung; **(J)** and kidney tissue sections. (**B-C, G, K-M**) Liver, lung and kidney function markers in serum (mean ± standard deviation of three separate experiments). (**D** and **H**) TNF-α in serum and BALF; (**E** and **I**) IL-1β in serum and BALF (mean ± standard deviation of three separate experiments). ** *p*<0.01 vs. the sham group, ^#^ *p*<0.05 vs. the MODS group, and ^##^*p*<0.01 vs. the MODS group.

No liver cell injury was detected in the sham group; however, rats with MODS presented with exhibited albuminoid degeneration and vacuolar degeneration in the liver in a time-dependent. Locally, eosinophilic variants and spotty liver necrosis were observed; phepatic sinus Kupffer cell proliferation and further inflammatory cell infiltration were also identified in the liver. However, only focal eosinophilic variants of liver cells were observed in the Nec-1 treatment group (Figure 5A).

These results were confirmed by assessment of liver function. In the Nec-1 treatment group, the levels of ALT and AST were significantly lower than in the MODS group at 4 hours and 8 hours (all *p*<0.01, Figure 5B and 5C). In addition, in Nec-1-treated animals, levels of the inflammatory cytokines TNF-α and IL-1β were significantly lower than in the MODS group at 4 hours and 8 hours (all *p*<0.05, Figure 5D and 5E).

### Nec-1 administration reduces necroptosis-induced lung injury

Similarly, no lung cell injury was observed in the sham group; while MODS rats exhibited alveolar edema complicated with hemorrhage and pulmonary interstitial edema complicated with focal inflammatory cell infiltration in a time-dependent. Alveolar septum thickening and interstitial pulmonary perivascular lymphocytic infiltration were also observed. In the Nec-1 treatment group, the lesions were markedly less pronounced, and pulmonary interstitial infiltration, and alveolar septum inflammatory cell infiltration were only occasionally visible (Figure 5F). Furthermore, Nec-1 treated animals had lower BALF, TNF-α, and IL-1β than MODS group animals at 12 hours and 24 hours (all *p*<0.05, Figure 5G-5I).

### Nec-1 administration reduces necroptosis-induced kidney injury

No kidney cell injury was observed in the sham group, while in the MODS group hyperemic and ischemic changes were observed in the glomeruli. Renal tubular epithelial cells exhibited albuminoid degeneration, and granular degeneration, and partial epithelial vacuolar degeneration was observed. Local interstitial perivascular edema and lymphocyte infiltration were also observed in a time-dependent. In the Nec-1 treatment group, only partial glomerular hyperemia and local renal tubular epithelial albuminoid degeneration were visible (Figure 5J).

These results were confirmed by assessment of kidney function. In the Nec-1 treatment group the tubular necrotic score, and levels of BUN, and SCR were significantly lower than in the MODS group at 12 hours and 24 hours (all *p*<0.01, Figure 5K-5M). Taken together, these results clearly demonstrate that Nec-1 reduced MODS-induced multiorgan injury and dysfunction.

### Nec-1 inhibits HMGB1 release and MLKL activation

To investigate the mechanism by which Nec-1 ameliorated multiorgan injury and dysfunction in MODS, we examined the effect of Nec-1 on critical substrates of necrosome formation; HMGB1 release and MLKL-pseudokinase (p-MLKL).

**Figure 6:**
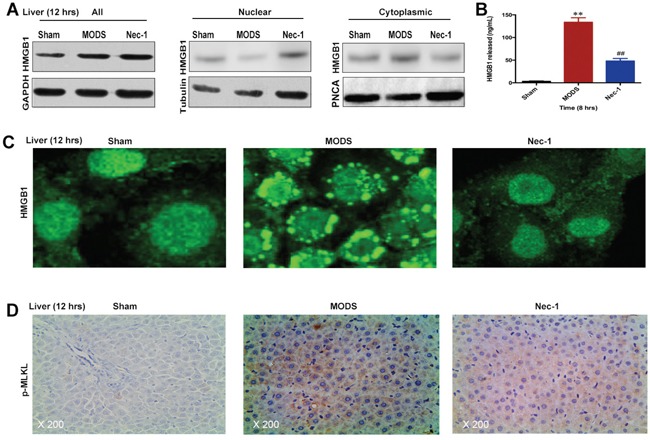
Nec-1 inhibits HMGB1 release and MLKL pseudokinase in rats with MODS (**A** and **C**) HMGB1 protein expression was evaluated in liver tissues by western blotting and IF; **(B)** HMGB1 release was evaluated by ELISA. **(D)** p-MLKL expression in liver tissues was assessed by IHC staining. Data shown were representative images (original magnification ×200) from three separate experiments. ** *p*<0.01 vs. the sham group and ^##^*p*<0.01 vs. the MODS group.

In sham rats, HMGB1 was predominantly located in the nucleus, whereas in MODS rats, higher amounts of HMGB1 were found to be translocated from the nucleus to the cytoplasm. Nec-1 significantly suppressed HMGB1 translocation from the nucleus to the cytoplasm at 12 hours (Figure 6A and 6C). Similarly, in MODS, release of HMGB1 into the serum was increased, while Nec-1 significantly suppressed HMGB1 release in MODS rats at 8 hours (*p*<0.05, Figure 6B).

In addition, we found that p-MLKL was strongly induced in MODS rats, while Nec-1 significantly suppressed p-MLKL in MODS rats at 12 hours (*p*<0.05, Figure 6D). These data suggest that Nec-1 treatment significantly inhibited HMGB1 release and MLKL pseudokinase.

## DISCUSSION

Despite significant improvements in the medical management of MODS, it is still associated with a high mortality rate. To our knowledge, this is the first report of the role of necroptosis in MODS. In the present study, our results indicate that necroptosis is involved the pathology of MODS, and Nec-1 exerted a marked treatment effect by attenuating multiorgan injury and dysfunction, significantly improving survival of MODS rats. This treatment effect was due primarily to suppression of necroptotic execution protein activation and inflammatory cytokine production. Therefore, a necroptotic inhibitor Nec-1 may be considered as an adjunct treatment in MODS.

Accumulating evidence indicates that necroptosis is involved in the regulation of lethal SIRS [11–14]. In this study, MODS-induced multiorgan necroptosis exceeded apoptosis, and was accompanied by necrosome formation. Necroptosis can be executed by necrosomes including RIP1, RIP3, and MLKL [19]. We found that RIP1, RIP3, and MLKL were expressed significantly more highly in the liver, lung and kidney of MODS animals than sham-group animals, while RIP1 expression did not differ significantly between two groups in the lung.

Next, we examined organ injury and dysfunction by monitoring serum levels of organ injury-associated enzymatic indicators including ALT, AST, and the level of proteins in BALF, tubular necrotic score, BUN, and SCR. We found that these enzymatic indicators were significantly elevated in MODS rats in comparison to the sham group. Interestingly, the time-dependent MODS-induced enzyme profile may indicate differences in the vulnerabilities of different organs to MODS. These indices of organ damage were significantly ameliorated by Nec-1 administration. In addition, the therapeutic effect of Nec-1 was also confirmed by histologic analysis. In liver, lung, and kidney samples, MODS-induced histopathologic changes, such as congestion, inflammatory cell infiltration, and degeneration, were ameliorated by Nec-1 administration. Although Nec-1 significantly reduced MODS-induced necroptosis, and organ damage, the function of several organs did not recover fully in Nec-1 treated rats. Therefore, early identification and immediate comprehensive treatment of MODS may improve outcomes.

The cellular mechanisms underlying the development and progression of MODS are complex, and of these multiple pathological processes the uncontrolled inflammatory response is known to contribute to rapid, progressive development of MODS [11–14]. Several studies have demonstrated that during early SIRS, the inflammatory cytokines TNF-α and IL-1β promote SIRS development [20–21]. Our results demonstrate that Nec-1 administration significantly inhibited MODS-induced elevations in TNF-α and IL-1β levels. Therefore, interfering with cytokine overproduction during early MODS may improve clinical outcomes. Importantly, Nec-1 also improved survival in MODS rats. In the MODS group, the survival rate was reduced from 85% to 65% 24 hours, and from 35% to 80% 48 hours, indicating that Nec-1 may be a potent and efficacious agent to treat MODS.

Measurement of the RIP3 substrate p-MLKL sheds light on the downstream mechanisms involved in executing necroptotic cell death [22–23]. We observed that p-MLKL was strongly induced in MODS rats, while Nec-1 significantly suppressed MLKL pseudokinase at 12 hours. In addition, necroptosis is thought to directly trigger inflammation through necrotic cells’ massive release of damage-associated molecular patterns, such as HMGB1 [24]. In the present study, HMGB1 was found to be translocated from the nucleus to the cytoplasm in MODS rats, while Nec-1 treatment significantly suppressed HMGB1 translocation and reduced HMGB1 release in serum at 8 hours. Taken together, these results suggested that Nec-1 significantly inhibited HMGB1 release and MLKL pseudokinase.

Our conclusions are, however, limited by the scope of this study. First, there is no broad consensus on the molecular mechanisms and timing of cell death following MODS. This is in part because few of the pathways that influence cell death are understood, and because cell death form is typically very hard to assess following MODS. Second, it is unclear how necroptosis differs from non-programmed forms of necrosis. Necroptosis is a broad term, and is yet to be precisely defined. The factors triggering necroptosis are largely unknown, as are the factors allowing cells to escape death via this mechanism. It is also unclear how other intracellular and extracellular factors may contribute to necroptosis.

Additionally, the NIH now requires both genders be represented in animal research in order to preclude sex hormones interfere with the data. In fact, an increasing body of evidence from animal models has revealed that sex hormones play an intricate role in the pathological response to trauma-hemorrhage [25–26]. However, clinical studies have been unable to consistently reproduce these laboratory findings [27–28]. Therefore, the sex based outcome differences post-injury remain conflicting. In this study, male and female MODS rat was manifested in significantly more severe multiorgan injury and multiorgan dysfunction than sham surgery, and Nec-1 exerted a marked treatment effect by attenuating multiorgan injury and dysfunction, significantly improving survival of MODS rats.

In conclusion, considering the emerging significance of necroptosis in inflammatory disease, a better understanding of the pathological processes underlying necroptotic signaling will likely have important implications for the development of novel inflammatory disease therapies. This is the first study showing that necroptosis is involved the pathology of MODS, and further the first to describe the use of a necroptotic inhibitor, Nec-1, to treat MODS. Nec-1 may in the future be considered as a candidate adjunct treatment for MODS.

## MATERIALS AND METHODS

### Ethics and statements

This study was carried out in strict accordance with the recommendations in the Guide for the Care and Use of Laboratory Animals of the National Institutes of Health. The experiment protocols was approved by our Institutional Animal Care and Use Committee and the independent ethics committee at Tianjin Medical University Cancer Institute & Hospital, National Clinical Research Center for Cancer, Tianjin, China.

### Animal and establishing trauma/hemorrhagic shock model

Six-week old male and female SD rats (200-250g), so that sex hormones would not interfere with the data, were obtained from the Experimental Animal Center, Academy of Military Medical Sciences, Beijing 100850, China (SCXK-2007-004). The rats were housed in a specific pathogen-free facility with free access to normal chow and water. The MODS rat model was successfully established as described previously [29–31]. Briefly, preconditioning was performed in a non-stressful, normothermic environment for more than 7 days prior to experiments. Rats were anesthetized by intraperitoneal injection of chloral hydrate (200 mg/kg). Afterward, a small incision was made, followed by catheterization in the right jugular vein and the right carotid artery for fluid resuscitation and bleeding, respectively.

An open mid-diaphyseal transverse fracture was created in the left femur to induce trauma, after which a mean arterial blood pressure of 30 ± 5 mmHg was maintained for 90 minutes through hemorrhage at 2.5mL/100g. Afterward, the rates were resuscitated for a period of 20 minutes with lactated Ringer's solution at a constant rate to manage the shock. The volume of the Ringer's solution was twice that of the blood loss. Finally, the catheters were removed, and the vessels were ligated. The mortality of experimental animals was about 30%.

### Treatment design

The experimental rats were randomly divided into three groups: (1) the sham group underwent the same anesthetic and surgical procedures and fluid resuscitation without induction of hemorrhage/trauma (n = 25); (2) the MODS group was described above (n = 25); and (3) the Nec-1 treatment group (n = 25), and the Nec-1 was purchased by Sigma, MO, USA, in which the MODS rats were pretreatment and once daily with Nec-1 (250 μg), which was diluted in resuscitation fluid and administered through the right jugular vein for about 20 minutes at a constant rate via a mini-pump. The dose of Nec-1 used in this study was based on previous study [11].

### Survival studies

The first endpoint was to determine the effect of Nec-1 treatment on survival from MODS; rats were randomly divided into three experimental groups as mentioned previously (n =25 per group). All rats had free access to water and food and were frequently monitored by dedicated research personnel to determine the 72 hours survival statistics.

### Fluorescence-activated cell sorting (FACS)

The Annexin V-FITC (BD Pharmingen, CA, USA) and PI binding assay were performed to determine the apoptosis and necroptosis of multiorgan tissue cells, and single-cell suspensions were prepared and stained as previously described [32]. The single-cell suspensions were trypsinized, washed in PBS, and resuspended in binding buffer. The cells were incubated in the dark for 10 minutes with Annexin V-FITC (100 ng/ml) and 10 ml PI added to each group. Positive Annexin V staining indicated apoptosis, while positive PI and Annexin-V negative indicated necroptosis (necrosis). For each group, 500,000 events for single-cell suspensions were acquired and the frequency of positive cells was measured. Then examined using flow cytometer (FCM, BD FACS Array, San Jose, USA).

### Protein extraction and western blotting (WB)

The multiorgan tissues were crushed in the dish, and the total, nucleus, and cytoplasm proteins were extracted by a cell lysate that was prepared containing an inhibitor cocktail against proteases and phosphatases. The protein concentration was measured by the BCA protein assay kit (Thermo Scientific, Rockford, Ill., USA). Equivalent amounts of protein were separated by 15% SDS-PAGE gel and transferred to a PVDF membrane at 4°C. The membrane was then incubated overnight with rabbit anti-mouse polyclonal RIP1 (#ab72139, abcam), RIP3 (#ab152130, abcam), MLKL (#5870, Sigma), high-mobility group box 1 (HMGB1,#ab18256, abcam), Tubulin (#ab6046, abcam), PNCA (#ab18197, abcam), and GAPDH (#ab8245, abcam, Cambridge, UK), respectively. Then, membranes were incubated with HRP-conjugated secondary antibody for one hour at room temperature. Antibody binding was detected using the electrochemiluminescence (ECL) detection kit to produce a chemiluminescence signal, which was captured on X-ray film. Relative protein expression levels were calculated relative to GAPDH.

### Enzyme-linked immunosorbent assay (ELISA)

The blood serum and bronchoalveolar lavage fluid (BALF) samples from rats were centrifuged at 3000 r/min for 5 minutes at 4°C, and the separated serum samples were stored at −20°C until use for assay. The ALT, AST, BUN, SCR, TNF-α, IL-1β, protein, and HMGB1 levels were measured using the monoclonal anti-rat capture antibody (R&D Systems, Minneapolis, MN, USA) according to the manufacturer's instructions. Calibration curves were established, and each microtiter plate with a standard curve. The colorimetric reaction was read with a Benchmark Microplate Reader (Benchmark Electronics, Inc., Angleton, TX, USA).

### HE staining and immunohistochemical (IHC) assay

The multiorgan tissues of rats from the different groups were collected and fixed with 10% neutral formaldehyde solution for 24 hours. The sample were dehydrated by a tissue-dehydrating machine, embedded in paraffin and sectioned into 4 μm thick slices. Following roasting, slicing and dewaxing, routine hematoxylin-eosin staining was conducted and the sections were observed under light microscopy using Olympus BX40F microscope (Olympus Melville, NY, USA). Images were captured with a Sony 3CCD color video camera (Sony, Tokyo, Japan).

The expression of p-MLKL in liver tissues of three experimental group rats were assessed by three independent researchers who were blinded to follow-up data. Their conclusions were in complete agreement for 85 % of cases indicating that this scoring method was highly reproducible. If two or all three agreed with the scoring results, the value was selected. If the results were completely different, then the pathologists worked collaboratively to confirm the score.

Briefly, the liver tissues from rats were dissected out, and tissue sections at 4 μm were rehydrated, treated with 3% H_2_O_2_ and blocked with 3% BSA. Subsequently, the tissue sections were incubated with anti-p-MLKL at Ser345 antibody (#ab208910, abcam, 1:500), or with control IgG overnight at 4°C. The bound antibodies were detected with HRP-conjugated second antibodies and DAB, followed by imaging under a light microscope. For evaluation of p-MLKL staining, a semi-quantitative scoring criterion was used, in which both staining intensity and positive areas were recorded. A staining index (values 0–16) obtained as the intensity of positive staining (week, 1; moderate low, 2; moderate high, 3; strong, 4) and the proportion of immune-positive cells of interest (0 %, 0; <10 %, 1; 10–50 %, 2; 51–80 %, 3; >80 %, 4) were calculated.

### Immunofluorescence (IF) staining

To confirm HMGB1 were translocated from the nucleus to the cytoplasm, we measured HMGB1 using IF staining. IF staining was briefly conducted on frozen section of liver tissues by HMGB1 (#ab18256, abcam, 1:500). The specimens were examined with an inverted fluorescence microscope (IX50, Olympus, Japan).

### Statistical analyses

Continuous variables were expressed as the mean ± standard deviation (SD), and these were compared using an unpaired Student's T-test and one-way ANOVA. Overall survival curves were estimated using Kaplan-Meier analysis and compared using the stratified log-rank test. The differences with *p*<0.05 (two-tailed) were considered statistically significant. Data were analyzed using the statistical software Intercooled Stata version 8.2 for Windows (Stata Corporation, College Station, Texas, USA).

## Abbreviations

DS: multiple organ dysfunction syndrome
Nec- 1: necrostatin-1
SD: Sprague-Dawley
SIRS: systemic inflammatory response syndrome
RIP1: receptor-interacting protein kinase 1
RIP3: receptor-interacting protein kinase 3
MLKL: mixed-lineage kinase domain-like protein
BALF: bronchoalveolar lavage fluid
TNF-α: tumor necrosis factor-α
IL-1β: interleukin-1β
ALT: alanine transaminase
AST: aspartate transaminase
BUN: blood urea nitrogen
SCR: serum creatinine
